# Child mental health in Jordanian orphanages: effect of placement change on behavior and caregiving

**DOI:** 10.1186/s12887-014-0316-1

**Published:** 2014-12-21

**Authors:** Michael J MacKenzie, Robin E Gearing, Craig S Schwalbe, Rawan W Ibrahim, Kathryne B Brewer, Rasha Al-Sharaihah

**Affiliations:** Columbia University, 1255 Amsterdam Ave., NY, NY USA; Columbia University Middle East Research Center – Amman, 1255 Amsterdam Ave., NY, NY USA

**Keywords:** Orphanage, Institutional care, Mental health, Behavioral problems, Internalizing, Externalizing, Goodness-of-fit, Placement change, Placement disruption

## Abstract

**Background:**

To assess the mental health and behavioral problems of children in institutional placements in Jordan to inform understanding of current needs, and to explore the effects of placement change on functioning and staff perceptions of goodness-of-fit.

**Methods:**

An assessment was completed of 134 children between 1.5–12 years-of-age residing in Jordanian orphanages. The Child Behavior Checklist was used to assess prevalence rates of problems across externalizing and internalizing behavior and DSM-IV oriented subscales. Also included was caregiver perceived goodness-of-fit with each child, caregiving behavior, and two placement change-clock variables; an adjustment clock measuring time since last move, and an anticipation clock measuring time to next move.

**Results:**

28% were in the clinical range for the internalizing domain on the CBCL, and 22% for the externalizing domain. The children also exhibited high levels of clinical range social problems, affective disorder, pervasive developmental disorder, and conduct problems. Internalizing problems were found to decrease with time in placement as children adjust to a prior move, whereas externalizing problems increased as the time to their next age-triggered move drew closer, highlighting the anticipatory effects of change. Both behavioral problems and the change clocks were predictive of staff perceptions of goodness-of-fit with the children under their care.

**Conclusions:**

These findings add to the evidence demonstrating the negative effects of orphanage rearing, and highlight the importance of the association between behavioral problems and child-caregiver relationship pathways including the timing of placement disruptions and staff perceptions of goodness-of-fit.

## Background

Despite a large and growing international literature underscoring the developmental and mental health deficits found in children reared in large institutional settings [1-5], many low- and middle-income countries continue to rely on orphanages as their sole or predominant model of care for children in out-of-home settings [6]. In the Middle East, children residing in institutional centers also exhibit similarly high rates of mental health difficulties, including youth in Jordan [7], Iraq [8], Turkey [9,10], and the Gaza Strip [6]. Institutionally-reared youth in the Middle East face the added challenge of aging-out into a collectivist society, where young adults without stable family relationships face substantial hurdles in accessing housing, establishing healthy social networks, obtaining employment, and succeeding in the marriage market [11].

The response in many countries to seminal work on institutional care such as the Bucharest Early Intervention Project [12], has been to point out that their institutions are not like the Romanian orphanages were, or to simply attempt to improve the orphanages by moving away from dormitories to smaller apartments with housemothers. It was within this context that the Jordanian Ministry of Social Development (MoSD) and United Nations Children’s Fund (UNICEF) partnered with the Community-Family Integration Teams (C-FIT) project group to develop community-based alternative care arrangements for children that would be acceptable to stakeholders in local communities. The C-FIT project has recently implemented the first pilot therapeutic foster care model in Jordan, with support from local judicial partners, NGOs, community leaders, and the Al Ifta council, which offers positions on the cultural and religious congruence of programs. As the nascent foster care program is established and available beds expanded, children continue to be cared for in institutions, including many older children for whom securing eventual foster placements will be more challenging. To this end, the C-FIT project in support with governmental agencies, NGOs and local community leaders sought in the current study to first establish the prevalence rates of early mental health and behavioral problems for children in institutional care in Jordan and to improve our understanding of how their trajectories through placements may exacerbate early deficits. Understanding these processes is important both to inform the design of foster care alternatives and to support efforts to strengthen institutional models to the extent possible for children remaining in center-based care.

Earlier research on behavioral and mental health of Jordanian adolescents in institutional care highlighted the impact of placement changes between institutions on child functioning [7]. The current study, with children spanning early childhood through the transition to adolescence, goes beyond examining the behavioral difficulties of the children to address three research questions. First, the study reports on the prevalence of emotional and behavioral difficulties in a sample of young children that reside in institutional settings in Jordan. Second, the study examines the impact of change and loss on child emotional and behavioral difficulties. We took advantage of planned placement changes that are scheduled according to the child’s age to calculate an “adjustment clock” (time since previous move) and an “anticipation clock” (time to next move). Finally, we examined the impact of behavior problems and placement change on staff reports of goodness-of-fit and links between these staff perceptions of fit and their caregiving behavior.

## Methods

### Study population and statistical analysis

This study is part of the Community-Family Integration Teams (C-FIT) project, a larger initiative aimed at assessing the mental health and developmental well-being of children in care homes in the Hashemite Kingdom of Jordan and developing community-based foster care alternatives to institutional placement. Cross-sectional survey and case file data were collected on all children between the ages of 18 months and 12 years in the selected age range residing in the three major care centers serving children in this age group across Jordan. Children enter into care homes through a variety of routes, including family disintegration, unwed pregnancy, child maltreatment, and as infants who were abandoned or from unknown parents. For each child, the primary staff member responsible for their care also completed a survey created by the investigators to assess the child’s emotional and behavioral problems as well as questions about caregiving, including caregiver perceived goodness-of-fit with the child, caregiving behavior items, and their expectations for the child’s future. In addition to the staff-report data, case files were reviewed to extract longitudinal data on reasons for placement, length of time in placement, and timing of any moves.

This study received approval from Institutional Review Boards at Columbia University and at the King Hussein Cancer Center in Amman Jordan for all procedures. Additionally, an independent Ph.D.-level Jordanian social worker served as a special advocate on behalf of the children to guard against the possibility that the Ministry, as official guardian of the children, might have an incentive for broad participation in order to increase their capacity to improve centers that might conflict with the needs of a particular child. The special advocate reviewed all study materials and questions and was provided the schedule for the study team’s visits to the care homes, so that he could perform unannounced site visits to observe our work and visit with children involved in any assessment. In the event of any special incidents, such as child distress during assessment, disclosure of maltreatment, disclosure of suicidal ideation, or concerns for child safety, the special advocate was also notified. Neither center directors nor Ministry officials were given information on whether staff or children participated in the surveys in order to protect ability to refuse participation. All staff completing surveys provided signed informed consent, and the Ministry serving as legal guardian of the children provided signed consent, via the center directors, for each child who was reported on by center staff in a survey.

### Measures

The first question to explore was the overall level of behavioral problems in the population of institutionalized children. The next question explored the association of children’s experience of placement change, both past and upcoming change, with measures of externalizing and internalizing behavior. Third, we employed hierarchical regression models to explore the association of caregiver perceptions of connection or fit with children they care for with measures of behavioral problems and children’s’ experience of placement change controlling for an array of child case characteristics. Finally, the measure of staff perceptions of goodness-of-fit was examined in bivariate associations with measures of caregiving expectations and behavior.

#### Mental health and behavioral functioning

Behavioral functioning and mental health were measured using the Child Behavioral Checklist (CBCL) Arabic language version completed by the child’s caregiver. The CBCL includes ratings for 113 behaviors on a three-point scale (0 = not true, 1 = sometimes true, 2 = very often true) [13,14], and has been previously used with Arabic speaking populations [15-17,7]. Respondents are instructed to consider the past six months in their ratings. The CBCL includes two major scales: internalizing problems and externalizing problems. These scales are further divided into subscales corresponding to an internalizing symptom cluster (e.g., Anxious/Depressed, Withdrawn/Depressed) and an externalizing symptom cluster (Aggressive Behavior and Rule Breaking in the version for 6–12 year-olds, and Aggressive Behavior and attention problems in the version for 1.5-5 year olds). The CBCL also includes subscales that correspond to DSM-IV diagnostic categories (e.g., affective disorder, anxiety disorder, pervasive developmental disorder, and conduct problems). The CBCL offers the advantage of normalized T-scores to allow for interpretation of scores with a normalized mean of 50 and a standard deviation of 10 points, and cut-points for borderline clinical and clinical range behavioral problems. Unfortunately, there are, to date, no community-based norms in Jordan.

#### Caregiver perception of Goodness-of-fit

Caregiver perceptions of fit with the child were assessed through a single item asking the caregiver “how good of a fit” they think that they have with the child, rated on a 5-point scale from Excellent (1) to Poor (5). The measure was translated and back-translated by Jordanian social service professionals and piloted on staff at other care homes.

#### Caregiving expectations and sensitivity/warmth

Caregiver expectations and behavior were assessed using three items. Staff were asked how far they would like to see the child go in school (1 = less than high school through 8 = doctoral degree). Caregiving sensitivity/warmth was assessed by asking the staff to report how often during the past month the caregiver had (a) spent time talking with the child about current events, and (b) spent time with the child doing one of their favorite activities (1 = not in the past month through 5 = every day).

#### Child characteristics and case factors

Case history data were extracted through a review of the case files, including: age, gender, reason for entry, length of stay, and whether a move had been experienced. The primary reason for entry was coded into the following three categories: maltreatment (e.g., neglect, physical, sexual or other abuse), family disintegration (e.g., parental divorce or imprisonment), and abandoned or orphaned. Length of stay at the care center was operationalized as the length of time in years between admission to the care center and the date of the study assessment. The length of time since the initial placement was calculated as the number of years between the first admission to a care center and the date of the study assessment. At the time of data collection, the policy of the care centers was to move children at certain age cut-offs, and these ages varied across different care homes. Based on this policy-induced variation across centers in the age when moves would be triggered, two time clock variables were calculated to approximate the amount of time since the child’s last move (adjustment to change clock) and the amount of time remaining until the child’s next move (anticipation of change clock).

## Results

134 children between the ages of 18 months and 12 years-of-age were enrolled in the study. The majority of the children in the care homes were male (57%) with a mean age of 7 years (S.D. = 3.3). Although all children in the sample had experienced at least one transition in caregiving upon their initial placement in the institutions, 41% had experienced at least one additional placement change between institutions since their initial movement into care. The mean length of stay in their current placement was 2.4 years, and the mean length of time in out-of-home care was 2.8 years. There was some diversity in regards to their pathway into out-of-home placement, with 47% placed because of family disintegration, 46% due to abandonment or being orphaned, and 7% as a result of child maltreatment.

The children evinced high levels of behavioral regulation problems as measured through the CBCL (Table 1). In the Total Behavioral Problems domain, over a third of the children were in at least the borderline clinical range or higher, with a quarter of the sample scoring in the clinical range. Nearly 40% of children exhibited at least borderline clinical internalizing problems, with 28% scoring in the clinical range. Through an examination of the syndrome scales that comprise the internalizing domain, we see that the highest levels of regulatory difficulty were in the anxious-depressed (10% clinical range) and withdrawn-depressed (22% clinical range) scales. Externalizing domain behavioral problem scores were also elevated, with nearly 30% scoring borderline clinical or higher, and 22% in the clinical range. Deficits were seen across all three of the syndrome scales, but the rule-breaking scale appeared as the greatest concern with 22% in the clinical range. Children also exhibited difficulties in sleep and thought problems with 6% and 5%, respectively, in the clinical range. Staff also reported high levels of social problems with one-quarter at borderline clinical or higher, with 17% in the clinical range.

**Table 1:** **Demographics, case history, and prevalence rates of behavioral and mental health problems on the CBCL syndrome scales, internalizing and externalizing domains, and DSM-IV diagnosis oriented scales in the Jordanian care home sample (n = 134)**

**Demographics and case history**	**%**	**Mean (SD)**		
Demographics				
Female	43%			
Age in years		7.0 (3.3)		
Case history				
At least one placement move experienced	41%			
Length of stay at current placement (years)		2.4 (2.3)		
Length of time in out-of-home care (years)		2.8 (2.8)		
Reason for entry				
Family disintegration	47%			
Abandoned or orphaned	46%			
Child maltreatment	7%			
**Child Behavioral Checklist (CBCL)**			**% Borderline clinical or above**	**% Clinical range**
**Total behavioral problems score**		55.3 (11.0)	34%	25%
**Internalizing domain**		55.8 (9.5)	39%	28%
Anxious-depressed		56.8 (6.9)	16%	10%
Withdrawn-depressed		61.1 (8.0)	34%	22%
Somatic complaints		53.7 (5.7)	9%	3%
Emotional reactivity^a^		56.1 (7.0)	20%	2%
**Externalizing domain**		53.5 (12.3)	29%	22%
Aggressive behavior		56.6 (8.8)	17%	8%
Rule-breaking^b^		59.9 (9.3)	27%	22%
Attention problems^c^		56.2 (6.8)	14%	6%
Sleeping problems^a^		54.0 (6.1)	6%	6%
Thought problems^b^		54.7 (6.3)	6%	5%
Social problems^b^		59.8 (8.1)	25%	17%
**DSM-oriented scales:**				
Affective disorder		59.4 (7.5)	21%	15%
Anxiety disorder		56.1 (7.0)	14%	7%
Pervasive developmental disorder^a^		61.7 (9.3)	44%	26%
Conduct problems^b^		61.6 (10.9)	36%	25%

^a^Scale only applies to ages 1.5 to 5 years old (n = 54); ^b^Scale only applies to ages 6 to 12 years old (n = 64); ^c^Attention is included as part of externalizing only for ages 1.5 to 5 years old.

On scales designed to map on to specific disorders of the DSM-IV, we also see high rates of problems emerging even in this pre-adolescent sample. For Affective Disorder, 21% of children scored in the borderline clinical range or higher, with 15% in the clinical range. For Anxiety Disorder, 14% of children were rated as in the borderline clinical or higher range, with 7% scoring in the clinical range. For the children from 1.5 to 5 years of age, 44% scored in the borderline clinical or higher range for Pervasive Developmental Disorder, with 26% in the clinical range. And for children from 6–12 years-of-age, 36% were in the borderline or higher range for Conduct Problems, with 25% in the clinical range. Across all CBCL scales, 42% of the children were in the clinical range on at least one, and 32% were in the clinical range on 2 or more scales.

**Figure 1:**
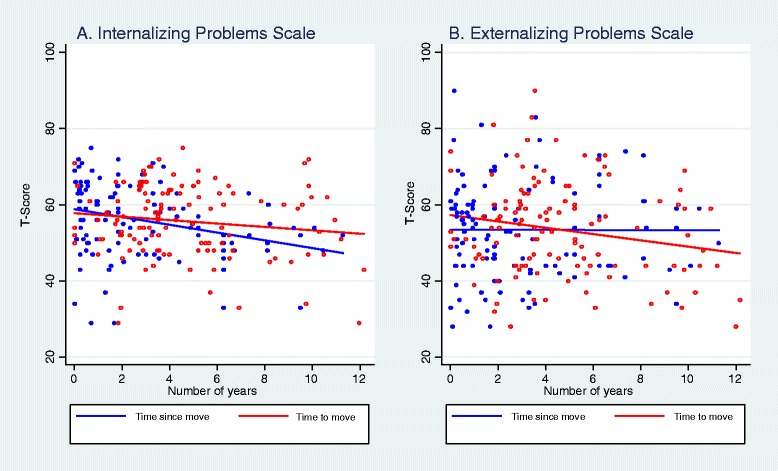
**CBCL T-Scores by placement change clocks for time until next move (anticipation clock) and time since last move (adjustment clock). A)** Internalizing behavior by years since last move (r(115) = −.31, *p* < .001) and years until next move (r(116) = −.14, *n.s.*). **B)** Externalizing behavior by years since last move (r(115) = −.01, *n.s.*) and years until next move (r(116) = −.20, *p* < .05).

We next explored the association between internalizing and externalizing problems and our placement change clock variables (Figure 1). We found that for internalizing problems (Figure 1A) there was an adjustment-effect of change, such that the clock measuring time since the child last moved was associated with significantly lower internalizing behavioral problem scores (r(115) = −.31, *p* < .001). For externalizing problems (Figure 1B), we found evidence for an anticipatory-effect of change, such that the clock counting down to measure time until the child’s next placement change was associated with increased externalizing behavior (r(116) = −.20, *p* < .05).

To better understand the associations between behavioral dysregulation and a child adjusting to past change or anticipating a coming change in placement, we next explored whether these processes (as measured by each child’s adjustment to change and anticipation of change clocks) were predictive of staff-reported goodness-of-fit or connection with the child (Table 2). In Model 1, we examined the potential contributions of internalizing behavior and the child’s adjustment to past change. We controlled for other potentially confounding factors such as gender, age, experience of prior moves, and reason for entry into out-of-home care, and found that increasing child age and internalizing behavior problems both significantly predicted poor goodness-of-fit with staff. The adjustment clock variable, however, remained significant in the model, such that as children adjusted to past change over time in a placement, they tended to find better fit with their caregivers. In Model 2, we examined externalizing behavior and the anticipatory effects of placement change as children draw closer to their next move, while controlling for the same set of variables as in Model 1. We found that externalizing behavior was predictive of poor caregiver-reported goodness-of-fit with the child and that, even after accounting for age, children who were closer to their next placement change were more likely to have a poor fit with their caregiver.

**Table 2:** **Association of child functioning and the anticipatory and adjustment effects of placement change on care home staff perceptions of relational goodness-of-fit with the child**

	**Staff reported poor Goodness-of-Fit with child**	
	**Model 1: Adjustment clock**	**Model 2: Anticipation clock**
Predictors	B (SE)	B (SE)
Intercept	.79 (.55)	.99 (.44)*
Female	.10 (.16)	.11 (.15)
Age	.05 (.03)*	-.03 (.02)
Prior move	-.11 (.16)	-.02 (.15)
Reason for entry *(Referant: abandoned/orphaned):*		
Family disintegration	-.17 (.19)	-.05 (.16)
Maltreatment	-.22 (.33)	.14 (.30)
Adjustment Clock (time since move in months)	-.01 (.003)*	--
Internalizing	.02 (.01)*	--
Anticipation Clock (time to next move in months)	--	-.01 (.002)*
Externalizing	--	.03 (.01)***
n	117	118
F(df)	2.04* (7, 109)	3.45** (7, 110)
R^2^	.12	.18

*p ≤ 0.05, **p ≤ 0.01, ***p ≤ 0.001.

Model 1 utilizes the Adjustment clock and internalizing domain scores on the CBCL to predict staff reports of goodness-of-fit with the child. Model 2 utilizes the Anticipation clock and externalizing domain scores to predict staff reports of goodness-of-fit with the child.

The importance of caregiver perception of goodness-of-fit, or connection with a child, was underscored by the association of these staff perceptions of fit with markers of caregiver expectations for the child’s future and caregiver warmth. Caregiver goodness-of-fit was associated with how much schooling the caregivers said they would like to see the child complete (r(127) = −.28, *p* < .01), indicating the importance of staff perceptions of fit to their expectations for the child. Perceptions of goodness-of-fit with the child also predicted staff caregiving behaviors, such as how often they spent time with the child in the past month doing one of the child’s favorite activities (r(128) = −.29, *p* < .001), or how often they spent time talking with the child (r(126) = −.17, *p* < .05).

## Discussion

The current study of institutionalized children in Jordan adds another layer of support for the growing global research literature highlighting the struggles faced by younger children reared in institutional settings [2,10,12,18]. The urgency of addressing this situation is underscored by the more severe mental health outcomes observed in adolescents in these settings [7,9,11,19], as the youth age through and out of the system. Earlier work with adolescents in Jordanian orphanages highlights the strong association between placement moves and functioning [7]. Here, capitalizing on the structure of the care home system wherein children know when their next age-triggered change will occur, we examine the adjustment and anticipatory effects of change from early childhood through the transition to adolescence.

Clinical range behavioral problems in at least one of the CBCL scales examined were observed in 42% of the children assessed, with levels of clinical range internalizing (28%) and externalizing (22%) scores comparable to those for youth in care centers in other countries [4-6]. High prevalence rates were also found across several DSM-IV related areas, most notably for the DSM-oriented scales of affective disorders (15%), pervasive developmental disorders (26%), and conduct problems (25%). Perhaps most disconcerting, and in keeping with work on psychosocial deprivation in early childhood in institutional settings [20,21] is the high rates of reported pervasive developmental disorder-oriented symptoms.

There is evidence that the increased problems evinced by older youth in care are not just a result of the quality of care in these settings, but also of the children’s experience of change and placement disruption as they age through and out of the system [7,20,21]. In older youth in Jordanian institutions, we found the number of prior placement moves to be an important predictor of mental health and wellbeing [7]. The younger children in this sample have not experienced as large a number of transitions at this point in their care trajectory, but the design of age-triggered moves in the orphanage system and the children’s awareness of when these changes will happen allowed us to explore both their adjustment to past change and anticipatory effects of upcoming changes. We find evidence of adjustment effects to change with regard to internalizing behavior, such that as the time increases since the child last moved we see decreases in internalizing behaviors. The opposite effect was found for externalizing disorder, such that as the length of time counts down until the child’s next move we see an associated increase in externalizing behaviors, highlighting the anticipatory effects of upcoming placement change.

The children’s adjustment to past change and anticipation of upcoming change, and the associated behavioral dysregulation, were also significant predictors of staff perceptions of the children and the extent to which staff felt they had a good-fit with a particular child. This anticipatory effect of change is in keeping with theoretical contributions around sensitivity to the prospect of rejection and defensive mechanisms in pushing people away [22]. This conceptual model of how children navigate coming change in placement finds support in the data showing that staff reports of how well they fit or connect with the child are impacted by the anticipation clock and the child’s externalizing behavior.

One potential limitation of the current study, however, is that we rely on staff report of child behavioral problems and staff perceptions of fit, which does not allow us to rule out the possibility that negative perceptions of the child have the potential to influence both the ratings of child behaviors and caregiver reports of fit. We remain confident, however, that caregiver bias does not account for the findings for two reasons. First, the CBCL asks about very specific child behaviors rather than just overall impressions of the child that would be more susceptible to bias from negative perceptions. Second, if negative caregiver perceptions of the child led to a generalized negative rating of the child that cut across different assessment constructs, then we would not have expected to find discrete associations for externalizing behavioral problems and internalizing behavioral problems. The differential association of adjustment to change and anticipation of change with internalizing and externalizing behaviors, provides evidence that caregivers were able to report on different domains of behavior in meaningful ways. The lack of community-based norms for the CBCL in Jordan also presents some limitation to be addressed in future work. Moving forward, we would also look to develop a broader scale of staff perceptions of goodness-of-fit. We don’t see this single-item measure as a major limitation, however, as the measure in interested in the staff’s perception of fit and staff demonstrated variation in their responses suggesting that they felt able to identify a range of children they did not fit well with and those with whom they fit better.

## Conclusions

The deficits for children in large institutions highlight the need for stable community-based alternatives to institutional care, but as those alternatives are implemented through recent reforms such as the C-FIT therapeutic foster care system in Jordan, there will continue to be a need to strengthen the institutions to the extent possible for children, particularly older children, likely to experience difficulty being placed. Taking up the charge of the seminal work of McCall and colleagues [21], the Jordanian Ministry of Social Development has undertaken commendable efforts to shift away from large dormitory style orphanages toward more family-like apartment style centers, and efforts have been recently put into place to attempt to reduce the number of gender-related age-triggered moves between institutions. These steps to address placement instability are critical to limiting these child behavioral repertoires and strategies for negotiating the stress of placement change becoming routinized as a stable strategy for managing relationships as children move through the orphanage system.

## Abbreviations

C-FIT: Community-Family Integration Teams
MoSD: Ministry of Social Development
CBCL: Child Behavior Checklist

## References

[1] Bos K, Zeanah CH, Fox NA, Drury SS, McLaughlin KA, Nelson CA (2011). Psychiatric outcomes in young children with a history of institutionalization. Harv Rev Psychiatry.

[2] Johnson DE, Miller LC, Iverson S, Thomas W, Franchino B, Dole K, Kiernan MT, Georgieff MK, Hostetter MK (1992). The health of children adopted from Romania. JAMA.

[3] Smyke AT, Koga SF, Johnson DE, Fox NA, Marshall PJ, Nelson CA, Zeanah CZ, & the BEIP Core Group: **The caregiving context in institution-reared and family-reared infants and toddlers in Romania.** *J Child Psychol Psychiatry* 2007, **48**(2):210–218. 10.1111/j.1469-7610.2006.01694.x 17300560

[4] Kjelsberg E, Nygren P (2004). The prevalence of emotional and behavioral problems in institutionalized childcare clients. Nord J Psychiatry.

[5] Schmid M, Goldbeck L, Nuetzel J, Fegert JM. Prevalence of mental disorders among adolescents in German youth welfare institutions. *Child & Adol Psychiatry and Mental Health.* 2008; 2. doi:10.1186/1753-2000-2-2 10.1186/1753-2000-2-2 PMC2262059 18226213

[6] Thabet L. Mental health problems among orphanage children in the Gaza Strip. *Adoption and Fostering.* 2007;31.

[7] Gearing RE, MacKenzie MJ, Schwalbe CS, Brewer KB, Ibrahim RW (2013). Prevalence of mental health and behavioral problems among adolescents in institutional care in Jordan. Psychiatr Serv.

[8] Ahmad A, Mohamad K (1996). The socioemotional development of orphans in orphanages and traditional foster care in Iraqi Kurdistan. Child Abuse Neg.

[9] Erol N, Simsek Z, Munir K (2010). Mental health of adolescents reared in institutional care in Turkey: challenges and hope in the twenty-first century. Eur Child Adol Psychiatry.

[10] Simsek Z, Erol N, Oztop D, Munir K (2007). Prevalence and predictors of emotional and behavioral problems reported by teachers among institutionally reared children and adolescents in Turkish orphanages compared with community controls. Children Youth Ser Rev.

[11] Ibrahim RW, Howe D (2011). The experience of Jordanian care leavers making the transition from residential care to adulthood: the influence of a patriarchal and collectivist culture. Children Youth Ser Rev.

[12] Nelson CA, Zeanah CH, Fox NA, Marshall PJ, Smyke AT, Guthrie  D (2007). Cognitive recovery in socially deprived young children: the Bucharest Early Intervention Project. Science.

[13] Achenbach TM (1991). Integrative Guide for the 1991 CBCL/4-18, YSR, and TRF Profiles.

[14] Achenbach TM, Rescorla LA (2001). Manual for the ASEBA School-Age: Forms & Profiles.

[15] Ivanova MY, Dobrean A, Dopfner M, Erol N, Fombonne E, Fonseca AC, Chen WJ (2007). Testing the 8-syndrome structure of the child behavior checklist in 30 societies. J Clin Child Adolesc Psychol.

[16] Loughry M, Ager A, Flouri E, Khamis V, Afana AH, Qouta S (2006). The impact of structured activities among Palestinian children in a time of conflict. J Child Psychol Psychiatry.

[17] Yunis F, Eapen V, Zoubeidi T, Yousef S (2007). Psychometric properties of the Child Behavior Checklist/2-3 in an Arab population. Psychol Rep.

[18] van IJzendoorn MH, Juffer F, Poelhuis CWK (2005). Adoption and cognitive development: a meta-analytic comparison of adopted and nonadopted children’s IQ and school performance. Psych Bul.

[19] Leite LC, Schmid PC (2004). Institutionalization and psychological suffering: notes on the mental health of institutionalized adolescents in Brazil. Transcultural Psych.

[20] Bos KJ, Zeanah CH, Smyke AT, Fox NA, Nelson CA (2010). Stereotypies in children with a history of early institutional care. Arch Pediatr Adolesc Med.

[21] McCall RB, van IJzendoorn MH, Juffer F, Groark CJ, Groza VK (Eds.) *Children without permanent parents: Research, practice and policy. Monographs of the Society for Research in Child Development.* 2011, **76**(4):ᅟ. Serial No. 301 10.1111/j.1540-5834.2011.00634.x PMC4088358 25018566

[22] Tucker DJ, MacKenzie MJ (2012). Attachment theory and change processes in foster care. Child Youth Serv Rev.

